# Dental Advertisements

**Published:** 1859-06

**Authors:** J. Richardson


### DENTAL ADVERTISEMENTS.

BY J. RICHARDSON.

HAPPILY Dental Science is not distracted by the multiplicity of conflicting systems that so eminently characterize medical practice, and we may congratulate ourselves that our young profession is unencumbered with either Allopathic, Homœpathic, Hydropathic, Eclectic or Thompsonian dentists.

But we are not entirely unaffected by some of the evils growing out of these divisions, for in dentistry, as in medicine, we have our quacks and pretenders. The most excusable and least offending of such, are those who, professing a knowledge of their art, are neither qualified by nature or education, to practice it with credit. Their sin is one of *ignorance* and *omission*, and while they are out of the reach of the ordinary means of correction, they can only be crushed out, if at all, by giving them, in the language of the late Prof. Townsend, "*a tremenduous letting alone.*"

But there is another class more worthy of the lash, and of which the profession, by a process of active medication, would do well to purge itself. They are professed *allies*, who, under the spurious assumption of a recognized brotherhood, compromise their own standing, and bring reproach upon associates, by practices held, by common consent, disreputable. Of this class, the *graduated* dentist is, of all others, the most pernicious and insufferable, because tainted with the three-fold offence of *treachery*, *ingratitude*, and *positive misdemeanor*.

Among those clothed at every Annual Commencement, with the degree of Doctors in Dental Surgery, there will be found now and then some weak-kneed recipient who, failing in the important qualities of *personal integrity* and *self-reliance*, will seek to bolster up his fortunes and provide against impending failure, by all the contemptible aids that address themselves to the ignorant and credulous. True, therefore, to his instincts, he incontinently betakes himself to the press, and *presto*, by virtue of cheap puffing on the part of the suborned editor of some obscure country newspaper, and a miraculous process of self-inflation, extending through half a mortal column, the new-fledged aspirant for public favors is transformed, with incredible celerity, into the full stature of a *finished operator.* He parades his diploma as a guarantee of *acquired skill and experience*, and with defiant front challenges *competition.*

Now, such effrontery is an indictable offence, and the party implicated confesses himself fairly amenable to the law for obtaining money under *false pretences.* But the greater offence is, that under the operation of a false impression conveyed in these editorials and advertisements, namely, that a diploma is a virtual endorsement of *matured skill and experience*, the public will hold such institution conferring the degree, accountable in aiding and abetting a swindle, if the graduate should prove, (as he is most likely to sooner or later, with such proclivities,) an imposter—a *graduated* imposter, if you please.

Now, a full course of collegiate instruction does not contemplate the conversion of a raw student into the matured practitioner, as these *fast* gentlemen would lead community to believe, and none are better informed than they of the fact than any such use of a degree is a flagrant prostitution of its import and intent. A diploma is but a simple attestation that the student, having complied with all the requirements of the institution and passed a satisfactory examination, is well grounded in a knowledge of the *theory* and *principles* of his art. To this extent, and no more, a school is responsible for the qualifications of its alumni. If, through lack of judgment in the application of those principles to the exigencies of practice, or through want of manipulative tact or skill, or from indolence in self-culture, or from professional degeneracy, the graduate fails in his functions as an operator, or in his deportment as a professional man, the odium should rest where it belongs—with *himself.* Any attempt, by direct or indirect means, to make his diploma subservient to the practice of professional knavery, is rank with bad faith and treachery to his Alma Mater, and confers the moral right upon all honest men to summarily "read him out." Nor do we imagine that a diploma is a thing to be hawked about and thrust into the face of every newspaper editor whose inclinations or interest may prompt him to puff the professional mendicant into fictitious and unnatural proportions.

In our judgment, professional advertising should be limited to a simple announcement of the practitioner's name, title, and place of business, for the reason that outside of this limit the practice gravitates with all the certainty of a natural law toward empiricism. It is, therefore, an *unsafe* practice, however cautiously employed, and the dental, like the medical profession, would do well to make it a standard of legitimacy.

We will take leave of this subject by appending a "card" and "editorial" which we clip from a late number of the "Peru (Indiana) *Republican,*" which we submit without comment, only remarking, that in view of the recent controversy between Drs. Dills and H. R. Smith, involving matters relating to the professional conduct of the former, this fresh evidence of Dr. D.'s proclivity to quackishness, charged upon him by Dr. Smith, receives additional confirmation in this deliberate repetition of the offence, and we can not resist the painful conviction that he is fast drifting, with his eyes open, into the list of *incurables:*

A CARD.

Doctor Dills would call special attention to three of the *latest improvements* in dentistry, which he has the honor of introducing into this community, viz: 1st, inserting artificial teeth on "Vulcanite;" 2d, inserting artificial teeth on "Cheoplasty;" 3d, extracting teeth by means of electro-magnetism, *without inflicting pain.*

Among the peculiar advantages which the vulcanized and cheoplastic styles of work possess over other methods practiced in mounting artificial teeth, might be mentioned, 1st, Cleanliness—the work, when finished, presenting not a single lodgement for extraneous matter; 2d, Practical usefulness and durability; 3d, Cheapness; 4th, Superior adaptation in *fit*, by means of which, success attends it under the most unfavorable circumstances; 5th, last, but not least, it is effectual in restoring faded beauty,

"Where the mouth grows wide, and the face falls in."

These are the advantages possessed alike by the Vulcanite and Cheoplasty; it can be said of the former that a set of teeth mounted on it are so light as to *float on water*. It is not intended, in enumerating the advantages of this style of work, to underrate other styles; on the contrary, each method of scientific practice in dentistry, has its own advantages; different cases call for different kinds of work, which fact will be duly recognized by Dr. Dills, and such work done as the case may call for. He does not believe that gold, used as a base for dental substitutes, will very soon be entirely superseded.

The *advantages* gained by the patient in having an offending tooth plucked out without pain is—nothing! but the comfort attending it is—everything! The Doctor, anticipating incredulity in regard to the palliating influence of electricity used in dental surgery, promises, in case the battery fails to accomplish what is claimed for it, that no charge will be made for its use.

Doctor Dills locates *permanently* in Peru, and his determination is to merit the continued patronage of his patients; on this ground he is sanguine of final success—in view of which he is ready to meet any opposition—or *competition*.

---

☞ The card of Dr. Dills, Surgeon Dentist, will be found in our columns to-day. The Doctor has purchased the dental instruments and apparatus of Dr. Forgerson, of this place, and becomes his successor. He is a graduate of the Ohio College of Dental Surgery, in Cincinnati, of which he was also resident dentist for some time. In addition to the advantages of a thorough scientific course (of which we have seen satisfactory attestations,) Dr. Dills has had the advantages of considerable practical experience in connection with those eminent in the profession. We also notice that Dr. Dill's name figures among the contributors to Dental Journals. We think this is a good location, and we bespeak for the Doctor a liberal patronage

